# Validation of the Child Development Card (KKA) as a growth and development tool for stunted and normal children in West Java, Indonesia

**DOI:** 10.1080/16549716.2025.2547440

**Published:** 2025-08-28

**Authors:** Ratna Jatnika, Hendriati Agustiani, Syauqiyyah Syahlaa

**Affiliations:** a Department of Psychology, Faculty of Psychology, Universitas Padjadjaran, Sumedang, Bandung, Indonesia; b Center for Psychometrics Study, Faculty of Psychology, Universitas Padjadjaran, Sumedang, Bandung, Indonesia

**Keywords:** Child development card (KKA), psychometric properties, Guttman coefficient, cronbach alpha, stunted, Indonesia

## Abstract

**Background:**

Stunting remains a major public health issue in Indonesia, and the limitations of anthropometric measures highlight the need for alternative tools such as the Child Development Card (KKA).

**Objectives:**

This study aimed to determine the validity and reliability of the KKA as a tool to measure the growth and development of stunted and normal children through direct observation.

**Methods:**

The revised KKA was administered to 262 children aged 13–60 months, including 174 normal and 88 stunted children, from three stunting-locus regencies in West Java Province, Indonesia. The data were analysed using content validity, the Guttman coefficient of reproducibility, and Cronbach alpha to develop a revised KKA observation guideline.

**Results:**

A total of 15 conceptually inappropriate items were revised, and an observation guideline was developed as a guide for the direct observation of children. In each aspect of growth and development, age range, and sample category, the reproducibility and scalability coefficients showed values of >0.9 and >0.6, respectively. Meanwhile, Cronbach alpha values for each age range and sample category were >0.7.

**Conclusion:**

The revised KKA demonstrated both validity and reliability as a tool for the detection, monitoring, and early intervention of growth and developmental delays in children with stunted as well as those with normal development.

## Paper Context

- **Main findings:** Developed a direct observation-based tool for assessing child development in both stunted and normal children.
- **Added knowledge:** Provided psychometric properties for a community-based developmental assessment tool tailored to the Indonesian context.
- **Global health impact for policy and action:** Offers a low-cost, comprehensive developmental assessment using simple household items, making it feasible for implementation in high-stunting prevalence areas.

## Background

Stunting is a significant public health problem worldwide, including in Indonesia. This condition refers to malnutrition in infants during the initial 1000 days of life, which leads to long-term effects that hinder both brain development and physical growth [1]. In addition, malnutrition during early childhood can impair physical development, inhibit mental growth, and potentially result in limited cognitive abilities in adulthood [2]. Early childhood development is critical, as it forms the foundation for school readiness, educational achievement, national productivity, and the development of social capital [3]. Children with stunting are at risk of reduced intellectual capacity, lower productivity, and an increased likelihood of developing degenerative diseases in the future [4].

According to the 2021 Study on the Status of Nutrition in Indonesia (*Survei Status Gizi Indonesia*, SSGI), the prevalence of stunting among Indonesian children was 24.4%. Although this reflects a decline compared to previous years, the National Medium-Term Development Plan (*Rencana Pembangunan Jangka Menengah Nasional*, RPJMN) has set a target to reduce this figure to 14% by 2024 [5]. In West Java, the prevalence of stunting remains high, increasing from 20.2% in 2022 to 21.7% in 2023, indicating a persistent public health concern [6].

Detection of stunting is commonly carried out using anthropometric measurements, such as height/length, weight, as well as arm and body circumference [7]. A child is classified as stunted when their height falls below minus two standard deviations from the growth standard median set by the World Health Organization (WHO) [8]. The conventional anthropometric definition is commonly applied to explain chronic malnutrition, and the concept is designed as a statistical indicator to measure the level of social and economic deprivation in children [9]. However, the absence of a clear biological basis for establishing the stunted threshold at −2 standard deviations (SDs) makes it a less precise classification for diagnosing malnutrition or individualised health conditions [10]. In this context, short physical stature alone may not accurately reflect underlying health or developmental problems [11]. Given these limitations in the detection of stunting, alternative approaches are needed to more comprehensively assess children’s conditions. One such approach is the evaluation of growth and development using tools like the Child Development Card, known in Indonesia as *Kartu Kembang Anak* (KKA).

The KKA is a tool used at Integrated Service Posts, known in Indonesia as *Pos Pelayanan Terpadu* (Posyandu), to monitor both parental caregiving activities and child development. It serves a dual function as a monitoring instrument and a communication aid, facilitating discussions on child development among healthcare providers, community health cadres at Posyandu, and mothers of young children [12]. The KKA has been developed by the National Population and Family Planning Agency (*Badan Kependudukan dan Keluarga Berencana Nasional*, BKKBN) since 1988. A user guide was published in 2010, and an online version of the tool was introduced in 2021. The KKA is designed for the early detection of developmental delays or disorders, covering seven domains: gross movement, fine movement, passive communication, active communication, intelligence, social behaviour, and self-help skills [12]. However, there is a lack of community participation in the use of the KKA.

Previous measurements of child development have relied on parent-reported data, which may be less accurate due to a dependence on memory recall rather than direct observation of a child’s behaviour at specific developmental stages. In contrast, assessments based on continuous, direct observation tend to have a lower risk of bias compared to other methods [13]. An earlier study by Colletta et al. [14] evaluated the reliability and validity of the original version of the instrument, reporting a reliability coefficient of 0.87 and a correlation of 0.91 with the Bayley Scales of Infant Development. The Bayley Scales [15] are administered by trained professionals and are known for their strong psychometric properties. However, their high cost of administration makes them less feasible for widespread use in community-based settings such as Posyandu. Similarly, the Cambodian Developmental Milestone Assessment Tool (cDMAT) [16], which evaluates domains including social/personal development, fine motor skills, language/cognition, and gross motor skills, offers a narrower scope. In response, the KKA was developed as a more comprehensive and contextually appropriate tool tailored to local community needs. Building upon the original validation findings, this study represents a revalidation effort, incorporating the necessary adaptations to enhance the tool’s relevance and applicability.

In the present study, the use of the KKA was modified to involve direct observation of children across key developmental domains. The instrument was refined and categorised according to various aspects of child growth and development. Observation guidelines were established for four age groups: 0–6 months, 7–12 months, 13–24 months, and 25–60 months. This grouping was implemented based on the understanding that developmental changes are particularly rapid and pronounced during the first year of life (0–6 and 7–12 months). After 12 months, developmental progress tends to slow, placing greater emphasis on the quality rather than the quantity of developmental milestones [17].

## Methods

### Variable

The importance of measuring child development using the KKA through direct observation underscores the need for rigorous validity and reliability assessments to ensure the accuracy and consistency of the instrument. In this context, validity was evaluated using content validity and Guttman coefficient of reproducibility. Content validity is a crucial parameter that ensures each item in the instrument accurately reflects the developmental dimensions being assessed, thereby confirming the instrument’s relevance and representativeness. Additionally, Guttman coefficient of reproducibility was employed to assess unidimensionality, evaluating the extent to which the KKA serves as an appropriate scale for measuring specific developmental aspects. This coefficient has also been utilised in previous studies to validate instruments such as The Sexual Sadism Scale (SeSaS) [18], Public Health Center Resilience [19], and The Distance Vision Scale [20]. Reliability, on the other hand, was measured using Cronbach alpha. This study contributes significantly to the literature by introducing a methodological approach for evaluating the KKA, particularly in the context of its psychometric properties.

This psychometric study aimed to examine the validity and reliability of the KKA, with the following measured variables:

1. Gross movement: an aspect of growth and development where children make movements including most of the body’s muscles and require energy.
2. Fine movements: made by certain parts of the body and only include a small number of muscles since children do not require much energy.
3. Intelligence: the child’s ability to capture, think, remember, and solve problems.
4. Passive communication: the child’s ability to understand gestures and speech from others.
5. Active communication: the child’s ability to express feelings, desires, and thoughts through crying, gestures, or words.
6. Social behaviour: the child’s ability to socialise with family members and also other people in the surrounding environment.
7. Self-help: the ability and skill to conduct simple activities related to the child’s daily life.

### Measurement tools

The KKA measurement tool, which covers seven aspects of child growth and development, is presented in Table 1.

**Table 1.** Child Development Card (KKA) items.

Age (Months)	Child Behavior	Development Aspect
1	Eyes glance to the right or left	Passive communication
2	Reply smiling at others	Social behavior
3	Tilt head	Gross movement
4	Self-tilt	Gross movement
5	Produce 3 different sounds	Active communication
6	Reach for and hold objects in front of them	Fine movement
7	Sit independently without assistance	Gross movement
8	Open the lid of the toy	Fine movement
9	Active in the game “Peekaboo”	Social behavior
10	Take objects with thumb & other fingers	Fine movement
11	Clapping, greetings, farewell, etc.	Intelligence
12	Walking close when called	Fine movement
13	Walk independently	Gross movement
14	Brew a drink with a spoon	Intelligence
15	Insert/remove small objects	Fine movement
16	Say 2 different words correctly	Active communication
17	Give 3 object by naming	Intelligence
18	Know and name the 3 parts of the body	Passive communication
19	Eat independently with a spoon	Self-help
20	Pronounce sentences consisting of two words	Active communication
21	Recognize 3 pictures and say the names	Passive communication
22	Arrange up 5 pieces of objects without falling	Intelligence
23	Saying when need to pee or defecate	Self-help
24	Kick the ball without holding on	Gross movement
25	Mention the names of 3 objects with their use	Active communication
26	Hand washing with shower	Self-help
27	Answering the question “What are you doing?”	Active communication
28	Standing on the toes of both feet	Gross movement
29	Draw straight lines correctly	Intelligence
30	Carry out 2 orders at once	Passive communication
31	Undress with buttons without assistance	Self-help
32	Collect similar objects	Intelligence
33	Use question or denial sentences	Active communication
34	Draw a circle with ends meeting	Intelligence
35	Say own name & gender	Active communication
36	Active socializing with friends	Social behavior
39	Draw various shapes	Intelligence
42	Fasten the cloth buttons correctly	Self-help
45	Mention yourself with “I”	Active communication
48	Separate objects of different sizes	Intelligence
51	Draw people	Intelligence
54	Cut food with a knife	Self-help
57	Carry out 3 sequential orders	Passive communication
60	Dance to music	Fine movement
63	Tell simple stories	Active communication
66	Count goods to 10	Intelligence

Table 1 shows that the KKA consists of 46 behavioural items distributed across seven developmental aspects. The instrument is administered monthly for each aspect of growth and development in children aged 1–36 months, and once every 3 months for children aged 39–66 months [1]. The measurements are conducted by asking parents, which may introduce bias, as the assessment is not performed directly on the children. This can lead to subjectivity in the results. To address this, the KKA was revised by evaluating the items and modifying the method to involve direct observation of children by trained cadres at Posyandu.

The grouping of KKA items based on the seven aspects of growth and development represents a unidimensional measurement scale, as shown in Table 2. According to Guttman [21,22], a unidimensional scale reflects the positioning of an individual within a perfect order based on the response to an object in line with the seven aspects. The presence of this scale implies that a child can only achieve a subsequent developmental item after mastering the previous one. Attainment of each developmental stage is thus considered a necessary prerequisite for progression to the next.

**Table 2.** Child Development Card (KKA) based on growth and development aspect.

Development Aspect	Age (Months)	Child Behavior
Fine Movement	6	Reach for and hold objects in front of them
	8	Open the lid of the toy
	10	Take objects with thumb & other fingers
	12	Walking close when called
	15	Insert/remove small objects
	60	Dance to music
Gross Movement	3	Tilt head
	4	Self-tilt
	7	Sit independently without assistance
	13	Walk independently
	24	Kick the ball without holding on
	28	Standing on the toes of both feet
Intelligence	11	Clapping, greetings, farewell, etc.
	14	Brew a drink with a spoon
	17	Give 3 objects by naming
	22	Arrange up to 5 pieces of objects without falling
	29	Draw straight lines correctly
	32	Collect similar object
	34	Draw a circle with ends meeting
	39	Draw various shapes
	48	Separates objects of different sizes
	51	Draw people
	66	Count goods to 10
Active Communication	5	Produce 3 different sounds
	16	Say 2 different words correctly
	20	Pronounce sentences consisting of two words
	25	Mention the names of 3 objects with their use
	27	Answering the question “What are you doing?”
	33	Use question or denial sentences
	35	Say own name & gender
	45	Mention yourself with “I”
	63	Tell simple stories
Passive Communication	1	Eyes glance to the right or left
	18	Know and name the 3 parts of the body
	21	Recognize 3 pictures and say the names
	30	Carry out 2 orders at once
	57	Carry out 3 sequential orders
Self-Help	19	Eat independently with a spoon
	23	Saying when need to pee or defecate
	26	Hand washing with shower
	31	Undress with buttons without assistance
	42	Fasten the cloth buttons correctly
	54	Cut food with a knife
Social Behavior	2	Reply smiling at others
	9	Active in the game “Peekaboo”
	36	Active socializing with friends

### Study sample

This study involved 262 children aged 13–60 months, consisting of 174 normal and 88 stunted children, selected from three regencies – Sumedang, Tasikmalaya, and Ciamis – in West Java Province. These three regencies were chosen from a total of 18 regencies in West Java, based on their status as designated stunting locus areas. Sampling was conducted using a cluster sampling method. In each selected regency, three to four villages were included. Within each Posyandu in villages, the number of stunted children was first identified, and for each stunted child, two normal children were selected to ensure adequate representation of the general population. The revised KKA instrument was tested only on children aged 13–24 and 25–60 months, as the measurable developmental aspects are most applicable within these age ranges.

### Data analysis

Data analysis was conducted using content validity and the Guttman coefficient of reproducibility. Content validity refers to the degree to which test items are appropriate to the test specifications and effectively represent the constructs being measured [23]. A test with good validity shows that the content is relevant and representative of the measured construct. In this study, validation included a detailed assessment of potential distortions in meaning caused by measurement or language issues, which could limit the interpretability of scores [24]. The evidence-based content of the KKA was established through an assessment of content validity conducted by three expert judges, including a professor and two researchers, with doctoral degrees specialising in parenting and child development. The coefficient of reproducibility (CR) was used as a criterion for assessing the validity of the unidimensional scale. This coefficient was calculated alongside other indicators, including minimum marginal reproducibility (MMR), percent improvement (PI), and the coefficient of scalability (CS). The minimum thresholds for the coefficients of reproducibility and scalability are presented in Table 3 [25].

**Table 3.** Reproducibility and scalability indicators.

Coefficient	Minimum Value
Coefficient of Reproducibility (CR)	>0.9
Coefficient of Scalability (CS)	>0.6

Data analysis was conducted by calculating the reliability of the instrument, as a reliable measurement tool is essential. This study employed Cronbach alpha as the reliability measure, with coefficients greater than 0.7 considered acceptable [26].

## Results

The results of the study regarding the demographics of the study sample are presented in Table 4.

**Table 4.** Demographics of the study sample.

Demographics	Normal	Stunted
Age 13–24 months		
n	44	22
Mean	18.77	18.77
SD	3.54	3.58
% Male	59%	59%
% Female	41%	41%
Age 25–60 months		
n	130	66
Mean	40.78	40.67
SD	9.86	8.85
% Male	48%	55%
% Female	52%	45%

The first stage involved conducting content validity on the KKA, carried out by three developmental psychology experts. The results indicated that several items were conceptually inappropriate and required revision, as shown in Table 5.

**Table 5.** Adjustment of Child Development Card (KKA) items.

Age (Months)	Child Behavior (KKA)	Development Aspect (KKA)	Child Behavior (KKA Revised)	Development Aspect (KKA Revised)
3	Tilt head	Gross movement	Lift head	Gross movement
7	Sit independently without assistance	Gross movement	Sit independently	Gross movement
9	Active in the game “Peekaboo”	Social behavior	Active in the game Peekaboo	Social behavior
10	Take object with thumb & other fingers	Fine movement	Take object with thumb & forefinger	Fine movement
11	Clapping, greetings, farewell, etc.	Intelligence	Clapping, greetings, farewell.	Intelligence
12	Walking close when called	Fine movement	Walking close when called	Passive communication
14	Brew a drink with a spoon	Intelligence	Stir a drink with a spoon	Fine movement
18	Know and name the 3 parts of the body	Passive communication	Know the names of 3 body parts	Passive communication
21	Recognize 3 pictures and say the names	Passive communication	Recognize 3 pictures	Passive communication
28	Standing on the toes of both feet	Gross movement	Standing upright on the tips of both feet	Gross movement
32	Collect similar objects	Intelligence	Collect objects with similar shapes and colors	Intelligence
39	Draw various shapes	Intelligence	Draw various simple geometric shapes	Intelligence
48	Separate object of different sizes	Intelligence	Separate objects of different sizes	Intelligence
60	Dance to music	Fine movement	Dance to music	Gross movement

Following the content validity assessment, the complete revised version of the KKA is presented in Table 6.

**Table 6.** The Child Development Card (KKA) revised entirely.

Development Aspect	Age (Months)	Child Behavior
Fine Movement	6	Reach for and hold objects in front of them
	8	Open the lid of the toy
	**10**	**Take object with thumb & forefinger**
	**14**	**Stir a drink with a spoon**
	15	Insert/remove small objects
Gross Movement	**3**	**Lift head**
	4	Self-tilt
	**7**	**Sit independently**
	13	Walk independently
	24	Kick the ball without holding on
	**28**	**Standing upright on the tips of both feet**
	**60**	**Dance to music**
Intelligence	**11**	**Clapping, greetings, farewell, etc.**
	17	Give 3 objects by naming
	22	Arrange up 5 pieces of objects without falling
	29	Draw straight lines correctly
	**32**	**Collect objects with similar shapes and colors**
	34	Draw a circle with ends meeting
	**39**	**Draw various simple geometric shapes**
	**48**	**Separate objects of different sizes**
	51	Draw people
Active Communication	5	Produce 3 different sounds
	16	Say 2 different words correctly
	20	Pronounce sentences consisting of two words
	25	Mention the names of 3 objects with their use
	27	Answering the question ‘What are you doing?’
	33	Use question or denial sentences
	35	Say own name & gender
	45	Mention yourself with ‘I’
Passive Communication	1	Eyes glance to the right or left
	**12**	**Walking close when called**
	**18**	**Know the names of 3 body parts**
	**21**	**Recognize 3 pictures**
	30	Carry out 2 orders at once
	57	Carry out 3 sequential orders
Self-Help	19	Eat independently with a spoon
	23	Saying when need to pee or defecate
	26	Hand washing with shower
	31	Undress with buttons without assistance
	42	Fasten the cloth buttons correctly
	54	Cut food with a knife
Social Behavior	2	Reply smiling at others
	**9**	**Active in the game Peekaboo**
	36	Active socializing with friend

Note: Items in bold were revised based on the results of the content validity evaluation.

Guidelines for the direct observation of children were developed based on age groups: 1–6, 7–12, 13–24, and 25–60 months, covering all aspects of growth and development. The upper age limit of 60 months was selected based on expert consensus that this age range represents the ‘golden period’ of development, peaking at 5 years before tapering off [17]. The observation guidelines used by cadres at Posyandu to assess children with the revised KKA include the following components:

1. Aspects of growth and development
2. Examples of behavior
3. Operational definitions of behavior
4. Required stimulation tools
5. Observation procedures
6. Instructions
7. Indicators of success

An example of an observation guide is presented in Table 7. The guide was developed with reference to Bentzen [27], which consists of instructions for the mother and child, indicators of success, intended behaviour, operational behaviour, necessary equipment, and measurement procedures for each behaviour.

**Table 7.** Sample observation guidelines.

**1 Month - Passive Communication**	
Behavior	Glances right and left.
Operational Behavior	The child glances to the right and left following the sound.
Tools	1. Yoga mat
	2. Rattles
Procedures	1. Ask the mother to put the child to sleep on her back.
	2. The examiner rings the rattle in front of the baby’s face, then moves it to the right and left.
	3. Make sure the child is not sleepy.
Instructions	No instructions are provided to avoid bias.
Indicators of Success	The child glanced right and left according to the sound (must be able to go left and right, both).
**8 Months - Fine Movement**	
Behavior	Opening the lid of a toy.
Operational Behavior	The child can hold the lid of the bucket and lift it.
Tools	1. Small closed bucket
	2. 3–5 small plastic balls
Procedures	1. The child is placed in a sitting position on a mat or flat surface.
	2. Place a closed bucket with 3–5 small plastic balls inside.
	3. The examiner models opening the lid of the bucket and shows that there are plastic balls inside.
	4. The child is asked to open the bucket lid to pick up the toy inside.
Instructions	“Let’s see what’s inside this bucket (modeling opening the lid of the bucket) Wow here, look there are toys (showing the ball inside the bucket to the child then closing it again). Let’s try to open it too.”
Indicators of Success	When the child can open the bucket lid containing the toy independently.
**16 Months - Active Communication**	
Behavior	Names 2 different words correctly.
Operational Behavior	A word consisting of 2 repeated syllables. Mostly with the vowel “a”.
Tools	–
Procedures	The examiner asks the child to name a word with repeated syllables, for example, Mama, Papa, Dada, Dede, and Mamam. Can say at least two words.
Instructions	“Come on, say Mama.”“Now say Papa.”And others according to the procedure.
Indicators of Success	It is said to be successful when the child can mention at least two words correctly according to the procedure (Mama, Papa, Dada, Dede, Mamam).
**60 Months - Gross Movement**	
Behavior	Dance to music.
Operational Behavior	Children follow gymnastic movements from 5 years old.
Tools	SKJ music on YouTube link: https://www.youtube.com/watch?v=a0vRs0a4rQQ
Procedures	The examiner shows the SKJ YouTube/music and then asks the child to follow the movements according to the video.
Instructions	“Let’s do gymnastics!”
Indicators of Success	The child can follow the movements according to the music.

Scoring was conducted by assigning a value of 1 if the child was observed to perform the expected behaviour, and 0 if the behaviour was not observed. Direct observations were carried out by trained cadres at the Posyandu, with the child’s mother present during the assessment process. Validity was assessed using the coefficient of reproducibility, as presented in Table 8.

**Table 8.** Validity of the Child Development Card (KKA) revised.

Category	Aspect Growth and development	CR	MMR	PI	CS
Age 13–24 Months					
Total	GK	1.00	0.96	0.04	1.00
	GH	0.99	0.93	0.06	0.91
	K	0.98	0.72	0.26	0.93
	KP	0.99	0.82	0.17	0.95
	KA	1.00	0.82	0.18	1.00
	TLS	1.00	0.97	0.03	1.00
	MDS	1.00	0.75	0.25	1.00
Normal	GK	1.00	0.96	0.04	1.00
	GH	1.00	0.95	0.05	1.00
	K	0.97	0.72	0.25	0.89
	KP	0.99	0.81	0.18	0.94
	KA	1.00	0.81	0.19	1.00
	TLS	1.00	1.00	0.00	1.00
	MDS	1.00	0.68	0.32	1.00
Stunted	GK	1.00	0.97	0.03	1.00
	GH	0.98	0.91	0.07	0.80
	K	1.00	0.73	0.27	1.00
	KP	1.00	0.84	0.16	1.00
	KA	1.00	0.83	0.17	1.00
	TLS	1.00	0.91	0.09	1.00
	MDS	1.00	0.75	0.25	1.00
Age 25–60 Months					
Total	GK	1.00	0.94	0.06	0.97
	GH	1.00	0.97	0.02	1.00
	K	1.00	0.80	0.19	0.99
	KP	1.00	0.92	0.08	0.98
	KA	1.00	0.81	0.18	0.99
	TLS	1.00	0.87	0.13	1.00
	MDS	1.00	0.82	0.18	0.99
Normal	GK	1.00	0.96	0.04	0.94
	GH	1.00	0.99	0.01	1.00
	K	1.00	0.81	0.17	0.99
	KP	1.00	0.95	0.05	1.00
	KA	1.00	0.84	0.15	0.99
	TLS	1.00	0.89	0.11	1.00
	MDS	1.00	0.82	0.18	0.99
Stunted	GK	1.00	0.91	0.09	1.00
	GH	1.00	0.94	0.06	1.00
	K	1.00	0.79	0.21	0.98
	KP	0.99	0.87	0.13	0.96
	KA	1.00	0.76	0.23	0.98
	TLS	1.00	0.84	0.16	1.00
	MDS	1.00	0.84	0.16	1.00

Description: GK = Gross Movement, GH = Fine Movement, K = Intelligence, KP = Passive Communication, KA = Active Communication, TLS = Social Behavior, MDS = Self Help. CR = Coefficient of Reproducibility, MMR = Minimum Marginal Reproducibility, PI = Percentage of Improvement, CS = Coefficient of Scalability.

Across all developmental aspects, age groups, and sample categories, the reproducibility and scalability coefficients exceeded 0.9 and 0.6, respectively. These results are considered excellent, as a reproducibility coefficient below 0.9 is not deemed sufficiently effective [25]. The reproducibility values also surpassed the minimum marginal reproducibility (MMR), indicating an acceptable level of reproducibility. In addition, the percentage of improvement (PI), which reflects the proportion of scale items requiring revision, was found to be low, suggesting that only minimal adjustments were necessary [25]. Following the validity assessment, reliability was evaluated using Cronbach alpha. Based on the results in Table 9, Cronbach alpha coefficients for each age group and sample category were above 0.7, indicating good internal consistency [26].

**Table 9.** Reliability of the Child Development Card (KKA) revised.

Category	Cronbach Alpha
Age 13–24 Months	
Total	0.80
Normal	0.81
Stunted	0.75
Age 25–60 Months	
Total	0.93
Normal	0.91
Stunted	0.93

## Discussion

This study aims to determine the validity and reliability of the KKA as a measurement tool for assessing the development of both stunted and normal children through direct observation. This study is the first to develop the KKA through direct observation and serves as a revalidation of the original version by Colletta et al. [14], ensuring its current relevance. Compared to previously available instruments, the Bayley Scales [15] demonstrate strong psychometric properties but are costly and less feasible for use in community-based settings such as Posyandu. Meanwhile, the Cambodian Developmental Milestone Assessment Tool (cDMAT) [16] assesses fewer developmental domains, which led to the refinement of the KKA into a more comprehensive and locally applicable instrument.

As shown in Table 5, 14 KKA items required adjustments based on the results of content validity conducted by three developmental psychologists. The revision process involved improving conceptual balance, item clarity, numbering, language, and diction, as well as refining the categorisation of developmental aspects. The revised KKA items are presented in Table 6, and the tool consists of 46 items that assess seven developmental aspects in children aged 1–60 months. The upper age limit of 60 months was selected based on expert consensus identifying the first 5 years of life as the ‘golden age’ of development, peaking before age five and gradually declining thereafter [17]. According to the 2020 Global Nutrition Report, stunting affects 140.9 million children under the age of five [28]. Children up to the age of five are also more vulnerable to morbidity and require significant care and attention [29].

The measurement of the seven developmental aspects in the revised KKA is supported by detailed observation guidelines, as illustrated in Table 7. This version adopts a direct observation approach to assess behaviours exhibited by children. These observation guidelines serve a dual function: evaluating the child’s development across each aspect and guiding parents in providing appropriate stimulation to support early detection of stunting. Parental involvement in stimulation is crucial [30], especially considering that children affected by stunted face increased risks for adverse outcomes such as impaired cognitive and learning development [2,31,32], delays in motor and language skills, and reduced socio-emotional competence [33,34] compared to normal children.

The revised KKA, now equipped with observation guidelines, demonstrates an excellent unidimensionality scale in measuring the seven developmental aspects. As such, the items can provide well-ordered developmental details from an early age to an older age. This result is reflected in Table 8, where the revised KKA shows excellent reproducibility (>0.9) and scalability (>0.6) coefficients.

The items in the revised KKA and the applied measurement methods demonstrate its effectiveness in evaluating children’s development. These findings align with previous studies showing that continuous, direct observation carries a lower risk of bias compared to other assessment methods [13]. Moreover, assessing children’s behaviour requires objective measurement, which is more accurate than subjective reporting [35]. This is particularly important given the challenges associated with memory accuracy when recalling the behaviours of young children [36], for which direct observation has been recognised as an objective and reliable method [37].

The validation of the unidimensional scale in the revised KKA provides a comprehensive representation of children’s developmental progression across the seven assessed aspects. Within a unidimensional framework, children are expected to master each developmental item sequentially – only after successfully completing the preceding milestone. Reaching one developmental stage serves as a prerequisite for progression to the next.

Reliability of the revised KKA was further confirmed through Cronbach alpha, with the results presented in Table 9. The instrument demonstrated strong internal consistency, consistently falling within the ‘good’ category, with alpha values exceeding 0.7 across all age groups and sample categories. These findings confirm the revised KKA as a reliable tool for evaluating developmental progress in both stunted and normal children using direct observation.

The results of this study indicate that the KKA can be applied in both stunted and normal populations. Its ability to capture developmental differences suggests that it is a suitable tool for routine use in community-based settings, such as Posyandu in Indonesia, to monitor child development across a broad range of nutritional statuses. Thus, the KKA demonstrates potential for use as a universal screening instrument to support early identification and intervention in both normal and stunted children.

The revised KKA adopts a direct observation approach to assess child development. In practice, observations can be carried out by trained community health workers at Posyandu (cadres) or by parents under cadre supervision. To support the practical implementation of the revised KKA, a structured observation manual has been developed, accompanied by guidance on the use of simple, household-based materials. The design of the tool intentionally minimises complexity to ensure its usability by cadres and parents in home or community settings. All assessment activities rely on readily available items, eliminating the need for specialised equipment. This approach enhances the feasibility and sustainability of developmental monitoring at the community level, particularly in resource-limited environments such as Posyandu.

Within community settings, these assessments can be integrated into existing platforms such as the Posyandu. When developmental delays are suspected, initial referrals may be directed to cadres. If further evaluation is required, cases can be escalated to professionals such as paediatricians or child psychologists. This tiered referral system promotes early detection and timely intervention within the local health infrastructure.

## Strengths and limitations

The KKA demonstrates strong validity and reliability as a direct observation tool for assessing early childhood development, and it is suitable for use in community-based settings, including for both stunted and normal children. Its use of simple, household-based materials makes it cost-effective and practical. However, successful implementation depends on adequate training of cadres or parents, who must understand basic developmental concepts. Individual differences among observers, particularly cadres, may still affect assessment accuracy despite training; therefore, regular refresher sessions are recommended, and involving more than one observer is advised. Broader-scale use will require further validation, and future dissemination of the observation guidelines through online platforms such as websites or YouTube may enhance accessibility and reach.

## Conclusion

In conclusion, the revised KKA is a valid and reliable measurement tool for assessing the development of both stunted and normal children. These findings are supported by the refinement of KKA items and the methodological shift to direct observation of children’s developmental behaviours. This revised instrument offers a more accurate means of assessing child development, particularly in identifying early signs of developmental delays. Furthermore, the confirmed validity and reliability of the revised KKA enhance its credibility as a developmental assessment tool. By incorporating item adjustments and adopting a direct observational approach, the KKA provides a more precise and contextually appropriate understanding of child development. Its implementation across various settings – including healthcare and early childhood education – can significantly contribute to early detection, timely intervention, and appropriate stimulation efforts. Greater attention and action in this area represent an important step towards preventing the long-term consequences of stunting and promoting optimal child development outcomes.

## Data Availability

Data that support the findings of the study will be available upon request from RJ, after seeking approval from the ethics committee.
